# Artificial intelligence compared with human-derived patient educational materials on cirrhosis

**DOI:** 10.1097/HC9.0000000000000367

**Published:** 2024-02-14

**Authors:** Faruq Pradhan, Alexandra Fiedler, Kaeli Samson, Marco Olivera-Martinez, Wuttiporn Manatsathit, Thoetchai Peeraphatdit

**Affiliations:** 1 Department of Gastroenterology and Hepatology, University of Nebraska Medicine, Omaha, Nebraska; 2 Department of Internal Medicine, University of Nebraska Medicine, Omaha, Nebraska; 3 Department of Biostatistics, College of Public Health, University of Nebraska Medical Center, Omaha, Nebraska

## Abstract

**Background:**

The study compared the readability, grade level, understandability, actionability, and accuracy of standard patient educational material against artificial intelligence chatbot-derived patient educational material regarding cirrhosis.

**Methods:**

An identical standardized phrase was used to generate patient educational materials on cirrhosis from 4 large language model-derived chatbots (ChatGPT, DocsGPT, Google Bard, and Bing Chat), and the outputs were compared against a pre-existing human-derived educational material (Epic). Objective scores for readability and grade level were determined using Flesch-Kincaid and Simple Measure of Gobbledygook scoring systems. 14 patients/caregivers and 8 transplant hepatologists were blinded and independently scored the materials on understandability and actionability and indicated whether they believed the material was human or artificial intelligence-generated. Understandability and actionability were determined using the Patient Education Materials Assessment Tool for Printable Materials. Transplant hepatologists also provided medical accuracy scores.

**Results:**

Most educational materials scored similarly in readability and grade level but were above the desired sixth-grade reading level. All educational materials were deemed understandable by both groups, while only the human-derived educational material (Epic) was considered actionable by both groups. No significant difference in perceived actionability or understandability among the educational materials was identified. Both groups poorly identified which materials were human-derived versus artificial intelligence-derived.

**Conclusions:**

Chatbot-derived patient educational materials have comparable readability, grade level, understandability, and accuracy to human-derived materials. Readability, grade level, and actionability may be appropriate targets for improvement across educational materials on cirrhosis. Chatbot-derived patient educational materials show promise, and further studies should assess their usefulness in clinical practice.

## Graphical abstract

**Figure:**
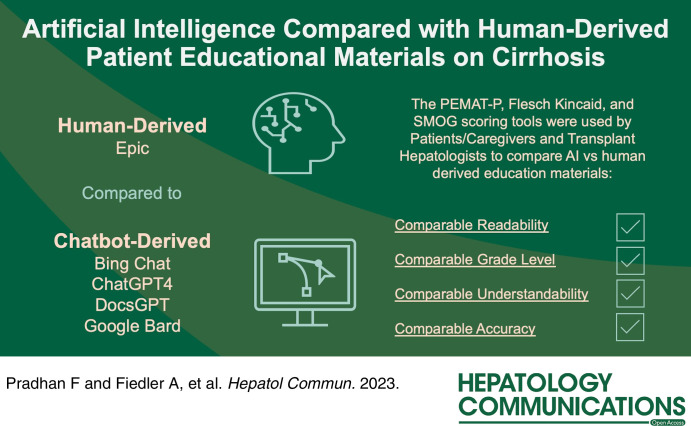

## INTRODUCTION

ChatGPT has been referred to as one of the fastest-growing apps of all time, reaching 100 million users within 64 days of being released in November 2022.^1,2^ This artificial intelligence (AI)-powered large language model chatbot is already being integrated into the Epic electronic health record system, which is a leading health care software provider in the United States, dominating nearly a third of the total market.^3^ ChatGPT has been groundbreaking in its unique interface, allowing it to engage the user in a conversation style that mirrors human dialogue. It has also shown promise in its ability to respond and interact in various languages.^4^

Given the rapid rise in chatbot use, there is an immediate need for further research exploring its use in different clinical applications.^5^ It is estimated that over half of adults in the United States use the Internet to acquire health information and advice.^6,7^ We postulate that patients are likely (or soon will be) using chatbots for this purpose as well.

We assessed the quality of chatbot-derived educational materials on cirrhosis, given its growing prevalence and complex management. Cirrhosis is the twelfth leading cause of death in the United States, causing over a million deaths annually.^8,9^ It confers a higher risk of mortality than many other chronic diseases, and early interventions may delay decompensation.^9,10^ Unfortunately, patients with cirrhosis often have insufficient knowledge about managing their disease and mitigating their risk for decompensation.^11^ The primary aim of this study was to assess the quality of educational materials on cirrhosis management obtained from various chatbots compared to human-derived educational material.

## METHODS

This descriptive study compared standard patient educational material to chatbot-derived patient educational material on cirrhosis. As this was a quality improvement project, it was exempt from institutional review board review. All patient educational materials were obtained on April 9, 2023, to maintain consistency. Human-derived standard educational material on cirrhosis was obtained from printable templates available from the Epic electronic medical record. AI-derived patient education material was obtained from 4 separate chatbots (ChatGPT, DocsGPT, Google Bard, and Bing Chat). The following standardized input phrase was used for all chatbots: “Compose a 1-page patient education sheet instructing patients about their diagnosis of cirrhosis and describe the complications of cirrhosis assuming the reader has a sixth-grade reading level.” This reading level was purposely selected based off American Medical Association (AMA) recommendations that all patient educational material be written at a sixth-grade reading level.^12–14^ To remove potential bias or adaptive responses to previously submitted queries, a “new chat” was initiated each time a prompt was submitted to each respective chatbot.

For uniformity of the materials, minor changes were permitted that included the removal of figures and graphs and standardization of the font and color; no changes were made to the content and no further adjustments to the formatting were permitted. The Bing chatbot required a conversational style to be selected, and “professional” was chosen.

All patient educational materials were scored in terms of readability and grade level, which measures how difficult a material is to comprehend. The 3 scoring systems used to assess readability and grade level are based off the complexity of the words and sentences used in the patient educational material and are calculated using previously validated formulas rather than subjective assessment by an evaluator. The Flesch-Kincaid Reading Ease tool was used to assess readability.^15^ Grade level was determined using 2 separate scoring systems: Simple Measure of Gobbledygook (SMOG)^16^ and Flesch-Kincaid^15^ grade level. The Flesch-Kincaid Reading Ease and Flesch-Kincaid grade level were determined using a readability statistics tool provided by Microsoft Word, while SMOG was determined using a calculator available online at https://charactercalculator.com/smog-readability.

The subjective assessment of the educational materials relied on the evaluation of the data by blinded evaluators consisting of 14 patients/caregivers and 8 transplant hepatologists. The 14 patients/caregivers were selected through convenience sampling; patients/caregivers in endoscopy waiting rooms were invited to participate in this study. There was no compensation for participation, and no subject identifiers were collected. Broad demographic data was collected and included the following: sex, age range, highest level of education completed, and English proficiency. The patients/caregivers were asked to self-determine their English proficiency using the predefined definitions provided by the US Department of State available at https://careers.state.gov/faq-items/what-are-the-language-proficiency-definitions/

All 22 evaluators were blinded to the material source and asked to independently assess each source for understandability and actionability using the Patient Education Materials Assessment Tool for Printable Materials scoring system.^17,18^ This scoring system includes 17 questions to assess understandability and 7 questions to assess actionability. However, since visual aids were removed and the fonts were standardized among the samples, questions 12–19 were eliminated as these were intended to assess the quality of visual aids. The options in response to each of the remaining questions included “Agree” (1 point), “Disagree” (0 points), and “Not applicable.” If “Not applicable” was selected, then the question was excluded when calculating the score. For our purposes, materials scoring ≥70% were considered understandable and actionable, as this cutoff has been used in prior studies.^19^

All evaluators were asked to state if they believed each educational material was created by a human, AI, or unknown (the evaluator is unsure if the sample was written by a human or AI). If the response “unknown” was selected by an evaluator, then it was excluded when calculating the percentage of evaluators who believed each educational material to be human-derived or AI-derived. The independent evaluators were not provided information regarding how many of the samples were chatbot-derived versus human-derived.

The patient educational materials were then assessed for accuracy of medical information by the 8 transplant hepatologists. Patients/caregivers were excluded from this aspect of the study. The 5-point scoring system used was adapted from *Dy* et al^20^ and *Storino* et al:^21^ a score of 1 indicates <25% is accurate, a score of 2 indicates 26%–50% is accurate, a score of 3 indicates 51%–75% is accurate, a score of 4 indicates 76%–99% is accurate, and a score of 5 indicates 100% is accurate.

Given the difference between the 2 types of raters in this study, scores from the transplant hepatologists and the patients/caregivers were analyzed separately and were not directly compared to each other. Descriptive statistics for continuous data were given as medians and IQR. Friedman tests were conducted to examine the difference in the distribution of scores between the educational material types. There was no data missing for any of the transplant hepatologists, as they were asked to address any missing data when submitting their responses. Conversely, missing data was present for the patients’/caregivers’ responses as they were collected anonymously, and therefore missing information was not later attainable. To assess the impact of the missing data, 2 analyses were therefore run: first using all available data, and second, as a sensitivity analysis, excluding all responses from patients/caregivers who had any substantial missing data (ie, were missing 2 or more items in calculating a score for at least 1 educational material type, n = 4). All statistical analyses were performed using SAS software version 9.4 (SAS Institute Inc., Cary, NC).

## RESULTS

Table 1 summarizes the objective scores obtained by previously validated scoring systems (Flesch Reading Ease, Flesch-Kincaid grade level, and SMOG grade levels) for each of the 5 materials. Based on the resultant Flesch Reading Ease scores, none of the materials were written at a level that someone with a sixth-grade education level would find comprehendible, as the minimal score requirement of 80 was not met. However, all 5 of the materials met the reading ease goal of ≥ 60, indicating someone with an eighth-grade education level would find the material comprehendible. Epic (human-derived) had the lowest Flesch Reading Ease score, indicating it was more difficult to comprehend. The Flesch-Kincaid grade levels ranged from 5.5–7.9, indicating all materials are comprehendible by someone with an eighth-grade education level.

**TABLE 1:** Objective scores for reading ease and grade level

	Epic (Human)	Bing Chat	ChatGPT4	DocsGPT	Google Bard
Flesch Reading Ease	60.2	63.7	72.3	72.6	63.5
Flesch-Kincaid Grade Level	7.4	7.9	5.5	5.7	7.5
SMOG Grade Level	11.6	12.3	9.4	12.1	14.3

*Note:* Average Flesch Reading Ease, Flesch-Kincaid grade level, and SMOG grade level using predefined calculations. Flesch Reading Ease scores ≥ 80 corresponded with the intended goal of materials being readable with a sixth-grade reading level and the higher the score indicates the easier it is to read. Flesch-Kincaid grade level and SMOG grade level is intended to be ≤ 6 indicating an individual with a sixth-grade reading level or above should be able to comprehend the material.

Abbreviation: SMOG, simple measure of gobbledygook.

The SMOG grade levels are also summarized in Table 1. Based off this scoring system, a person with a high school education or above is expected to be capable of successfully reading any of these educational materials. This scoring system suggests that the Google Bard-derived educational material is the most difficult to read and that an undergraduate education level may be needed to comprehend it.

Subjective scores for understandability, actionability, and accuracy were determined by patients/caregivers and transplant hepatologists. Broad demographic information was obtained for the patients/caregivers that provided demographic information (n = 12). Most were female (92.9%), and age frequencies were as follows: 8.3% ages 18–24, 25% ages 25–34, 16.7% ages 35–44, 41.7% ages 45–54, and 8.3% ≥ 55 years old. Education levels varied, with 41.7% having completed high school, 50% having an associate or bachelor’s degree, and 8.3% having a masters, PhD, or a professional degree. The average English proficiency level was 4.5 out of 5.

The median and IQR for understandability and actionability of each educational material for both patients/caregivers and transplant hepatologists are summarized in Table 2. Friedman tests were performed and showed no significant difference in the distribution of scores for understandability or actionability across the 5 educational material types for either patients/caregivers or transplant hepatologists (p > 0.05).

**TABLE 2:** All participants’ and transplant hepatologists’ subjective scores for understandability, actionability, and suspected author

	Understandability scores	Actionability scores	Suspected author	
	Median (IQR) (%)	Median (IQR) (%)	AI (%)	Human (%)
Epic (Human)				
Patients/caregivers	90.5 (81.8, 90.9)	77.5 (60.0, 80.0)	44.4	55.6
Hepatologists	90.5 (80.0, 90.9)	80.0 (50.0, 80.0)	50.0	50.0
Bing Chat				
Patients/Caregivers	90.9 (90.0, 100.0)	80.0 (80.0, 83.3)	60.0	40.0
Hepatologists	80.0 (70.0, 88.2)	60.0 (55.0, 90.0)	66.7	33.3
ChatGPT4				
Patients/Caregivers	95.5 (90.0, 100.0)	80.0 (71.4, 100.0)	33.3	66.7
Hepatologists	90.5 (90.0, 100.0)	63.3 (55.0, 83.3)	50.0	50.0
DocsGPT				
Patients/Caregivers	90.0 (80.0, 90.0)	65.7 (60.0, 80.0)	66.7	33.3
Hepatologists	85.0 (65.0, 90.5)	50.0 (20.0, 63.3)	80.0	20.0
Google Bard				
Patients/Caregivers	100.0 (90.0, 100.0)	80.0 (60.0, 85.7)	30.0	70.0
Hepatologists	85.0 (80.0, 95.0)	50.0 (50.0, 80.0)	33.3	66.7

*Notes:* All (including participants with > 1 missing response for ≥ 1 author) patients’/caregivers’ data (n = 14) and transplant hepatologists’ data (n = 8) regarding PEMAT-P scores for understandability and actionability are provided for each source as medians (%) and IQR (%). Materials scoring ≥ 70% on understandability and actionability are considered understandable or actionable. There was no significant difference in patients’/caregivers’ nor transplant hepatologists’ perceived understanding or actionability scores between authors. The percentage of patients/caregivers and transplant hepatologists identifying each source as written by a human or AI is also included.

Abbreviations: AI, artificial intelligence; PEMAT-*P*, patient educational materials assessment tool for printable materials.

As shown in Table 2, both groups scored all materials higher in understandability than actionability. All patient educational materials met the predetermined understandability score cutoff of ≥70%; therefore, all materials were considered understandable. The median scores for understandability determined by the transplant hepatologists were consistently lower than those determined by the patients/caregivers for all 5 patient educational materials. The human-derived material (Epic) was the only educational material to meet the predetermined median score cutoff of ≥70% to be considered actionable by both the transplant hepatologists (80.0%) and patients/caregivers (77.5%). Conversely, DocsGPT was the only source not considered actionable by either the transplant hepatologists (50.0%) or the patients/caregivers (65.7%).

The proportion of evaluators who thought each educational material type was written by a human versus AI is included in Table 2. Google Bard was most often suspected to be human-derived by both patients/caregivers (70.0%) and transplant hepatologists (66.7%). Conversely, DocsGPT was most frequently suspected to be AI-derived by both patients/caregivers (66.7%) and transplant hepatologists (80.0%). The overall data supports that the evaluators could not tell which patient educational materials were human-derived versus AI-derived.

To account for missing data for the patients/caregivers, a separate sensitivity analysis was conducted where participants with missing data were excluded from the analysis. The medians and IQR provided by the sensitivity analysis were similar, and the Friedman tests demonstrated no significant differences in the distribution of the patients’/caregivers’ perceived scores across the 5 educational material types for understandability and actionability. The corresponding data from the sensitivity analysis is included in the Supplemental Table S3, http://links.lww.com/HC9/A768.

**FIGURE 1:**
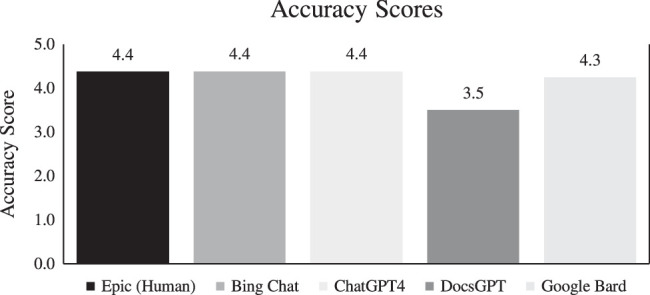
Transplant hepatologists’ average accuracy scores for each author based on the scoring system utilized in Dy et al^20^ and Storino et al.^21^ A score of 1 indicates < 25% of the information is accurate; a score of 2 indicates 26%–50% of the information is accurate; a score of 3 indicates 51%–75% of the information is accurate; a score of 4 indicates 76%–99% of the information is accurate; a score of 5 indicates 100% of the information is accurate.

Medical accuracy scores were only determined by the transplant hepatologists, which are summarized in Figure 1. The only material type to score below a 4 was DocsGPT, which had a score of 3.5. The human-derived and the other 3 chatbot-derived patient educational materials were considered to contain at least 76%–99% accurate information (scores above 4).

## DISCUSSION

The average American reads at an eighth-grade education level, and multiple health organizations, including the American Medical Association and National Institutes of Health have recommended that educational materials be written at no greater than the sixth-grade level.^12–14^ Despite this knowledge, the human-derived patient educational material (Epic) was estimated to be written at a seventh-grade level per the Flesch-Kincaid grade level and at the high school level per the SMOG reading level. Several of the chatbot-derived educational materials were also written above the sixth-grade education level; however, ChatGPT scored the lowest in the Flesch-Kincaid and SMOG grade levels. Therefore, ChatGPT was closest to the intended goal of being readable with a sixth-grade reading level and performed better than Epic (human-derived) at this. This goal is especially important in this patient population as prior studies have already demonstrated low health literacy in patients with cirrhosis.^22,23^ This shows that chatbot-derived educational materials may prove to be useful in generating future patient educational materials. Chatbots could be used to easily develop templates for educational materials that may then be edited by human authors.

Most materials met the predetermined Patient Education Materials Assessment Tool for Printable Materials scores for understandability and actionability, and Friedman tests showed there was no significant difference in the patients’/caregivers’ or hepatologists’ perceived actionability or understandability among the educational materials. Still, the actionability scores were consistently lower than the understandability scores, suggesting emphasis should be placed on improving the actionability across patient educational materials on cirrhosis. Additionally, most education materials scored well in terms of medical accuracy. This suggests that chatbots could be useful in creating templates of educational material that can then be assessed by a human for accuracy.

Although Google Bard was most frequently suspected to be human-derived and DocsGPT the most frequently suspected to be AI-derived, the overall data supports that the evaluators could not accurately tell if materials were written by human or AI authors. This is further supported by the fact that 7/20 (35%) of evaluators who participated in this portion of the survey selected unknown for at least one of the educational material types. This may suggest that AI chatbots are becoming increasingly more human-like in conversation style. However, the investigators were not asked to provide reasoning for why they suspected the author to be human or AI for each educational material type. Therefore, it remains unclear why evaluators suspected a material to be human-derived or AI-derived. It could be beneficial in future studies to have the evaluators comment on the reasoning behind their response. Although the use of chatbots has promising medical applications, there are identifiable limitations to their use. The chatbots used are not specifically intended for medical use and have the potential to generate inaccurate information or cite sources that do not exist.^4^ Chatbots respond in real time and are not vetted by a human before the recipient receiving their responses. Therefore, they should not serve as a substitute for or be developed without the help of an appropriate health care professional. Furthermore, current widely available chatbots are not capable of incorporating appropriate visual aids. Given patient educational materials often incorporate visual aids, this may be a limitation in using chatbots to create patient educational materials. However, the text-based portion may still be generatable by chatbots, and visual aids later incorporated by a human author. Furthermore, visual aids are used to make text more understandable and if chatbots can be used for this purpose (ie, develop educational materials at an appropriate grade level), then visual aids may not be necessary. This study has identifiable limitations as well. Since this study used a convenience sample to recruit patients/caregivers, the results may not be generalizable to the entire population. The chatbot input phrase requested the material be written at a sixth-grade educational level, but an actual chatbot user may be unlikely to specify this. The study results suggested that even when this is specified, some chatbot outputs can still be above the desired reading level. This further highlights the need for improvements in developing patient educational materials better suited for all patients, including those with lower educational levels and/or English proficiency.

## CONCLUSION

Overall, this study suggests that chatbots may have promising uses in patient care delivery. In general, the human-derived patient educational material did not consistently outperform chatbot-derived educational material, and users could not readily distinguish between human-derived and AI-derived educational materials. This study also highlights the need for further improvements in readability, grade level, and actionability across cirrhosis educational materials. Further studies are needed before AI chatbots can be readily adopted for routine clinical application.

## Supplementary Material
