# Supplementary material for: Artificial intelligence compared with human-derived patient educational materials on cirrhosis
Source: Hepatol Commun. 2024 Feb 14;8(3):e0367. doi: 10.1097/HC9.0000000000000367 (PMC10871753; doi:10.1097/HC9.0000000000000367)
Supplement: Supplementary file 1 [file hc9-8-e0367-s001.doc]

Table 3. Participants' Subjective Scores for Understandability, Actionability, and Suspected Author

		Understandability Scores	Actionability Scores	Suspected Author	
Author		Median (IQR) (%)	Median (IQR) (%)	AI (%)	Human (%)
Epic (Human)	Patients/ Caregivers	90.5 (81.8, 90.9)	77.5 (60.0, 80.0)	44.4%	55.6%
	Sensitivity Analysis	90.0 (80.0, 90.9)	73.2 (60.0, 80.0)	50.0%	50.0%
Bing Chat	Patients/ Caregivers	90.9 (90.0, 100.0)	80.0 (80.0, 83.3)	60.0%	40.0%
	Sensitivity Analysis	90.0 (88.9, 100.0)	80.0 (71.4, 80.0)	50.0%	50.0%
ChatGPT4	Patients/ Caregivers	95.5 (90.0, 100.0)	80.0 (71.4, 100.0)	33.3%	66.7%
	Sensitivity Analysis	90.9 (90.0, 100.0)	77.5 (60.0, 85.7)	20.0%	80.0%
DocsGPT	Patients/ Caregivers	90.0 (80.0, 90.0)	65.7 (60.0, 80.0)	66.7%	33.3%
	Sensitivity Analysis	90.0 (87.5, 90.0)	60.0 (60.0, 71.4)	60.0%	40.0%
Google Bard	Patients/ Caregivers	100.0 (90.0, 100.0)	80.0 (60.0, 85.7)	30.0%	70.0%
	Sensitivity Analysis	100.0 (90.9, 100.0)	60.0 (60.0, 80.0)	33.3%	66.7%

Two separate analyses of participant data were performed to account for missing data. The horizontal rows labeled "patients/caregivers" include all corresponding data for all patients/caregivers (n=14), while the horizontal rows labeled "sensitivity analysis" excludes any patients/caregivers with >1 missing response for any author (total n=10). PEMAT-P scores for understandability and actionability are provided for each source as medians (%) and IQR (%). Materials scoring ≥ 70% on understandability and actionability are considered understandable or actionable. There was no significant difference in patients'/caregivers' perceived understanding or actionability scores between authors, in either analysis. The percentage of patients/caregivers identifying each source as written by a human or AI is also included.

PEMAT-P= Patient Educational materials assessment tool for printable materials
